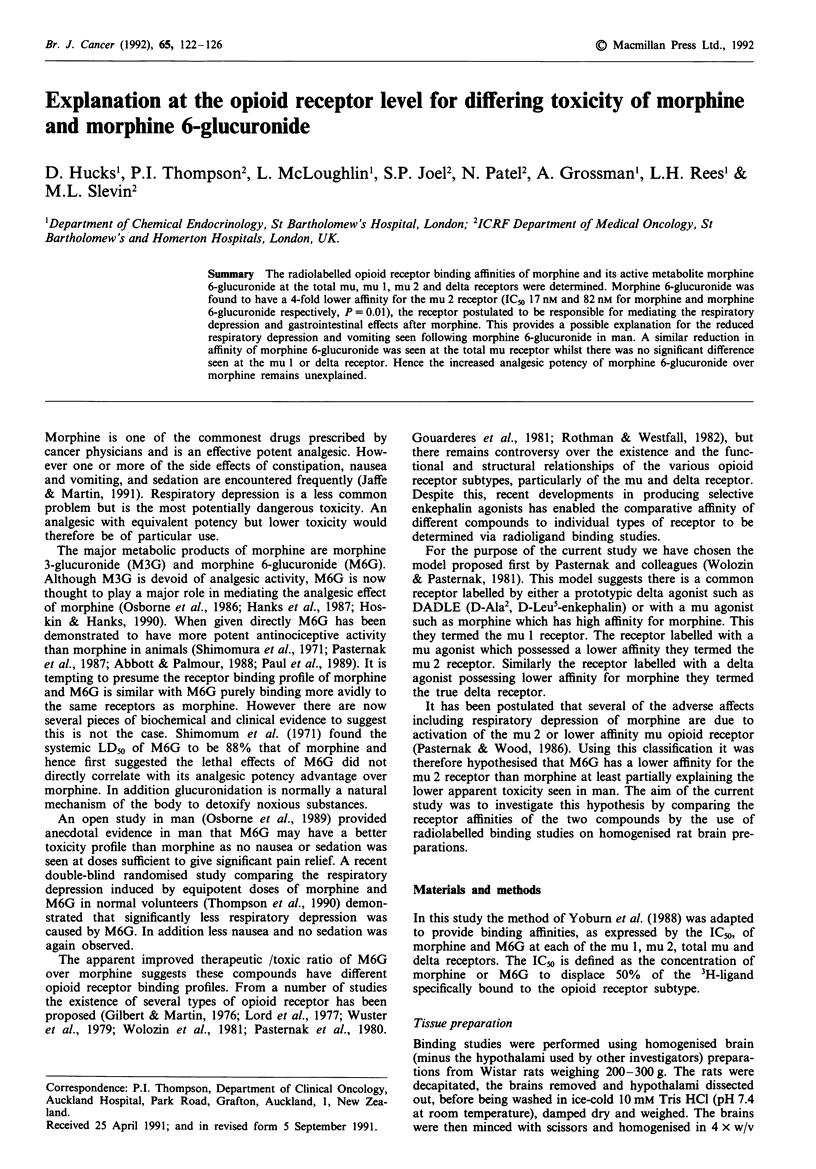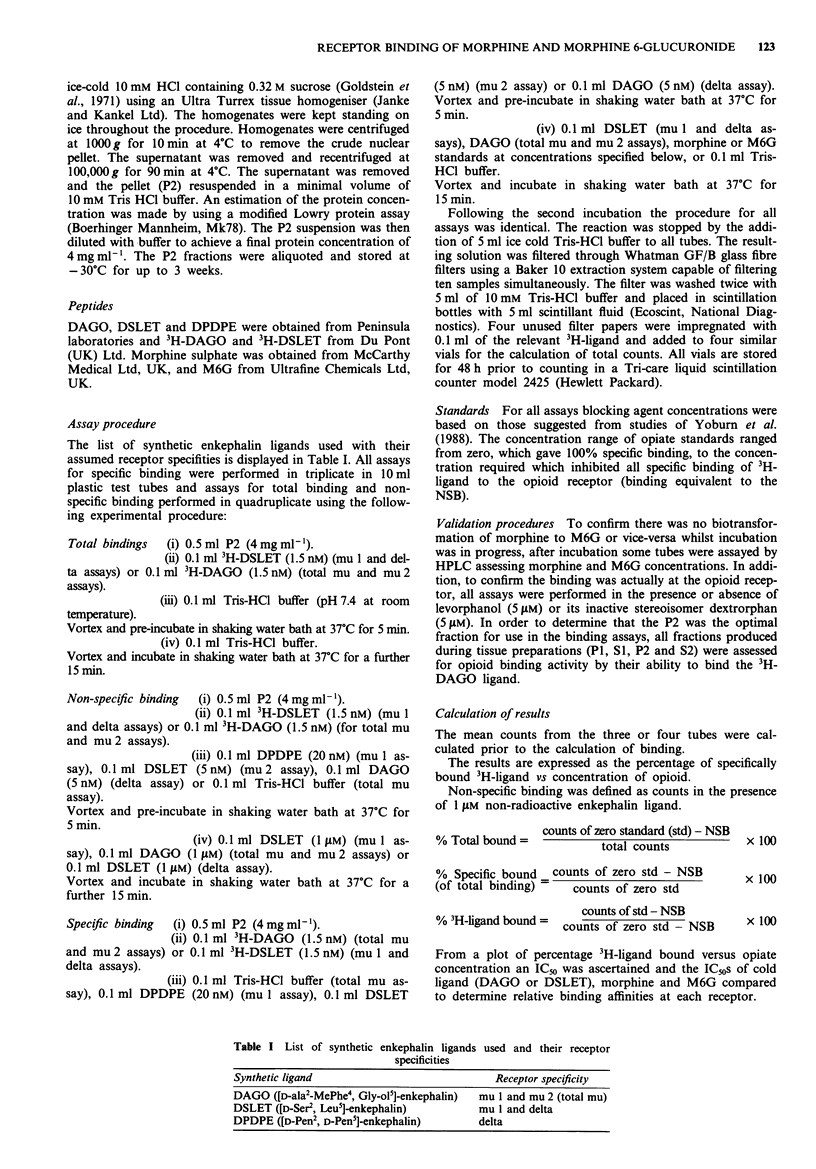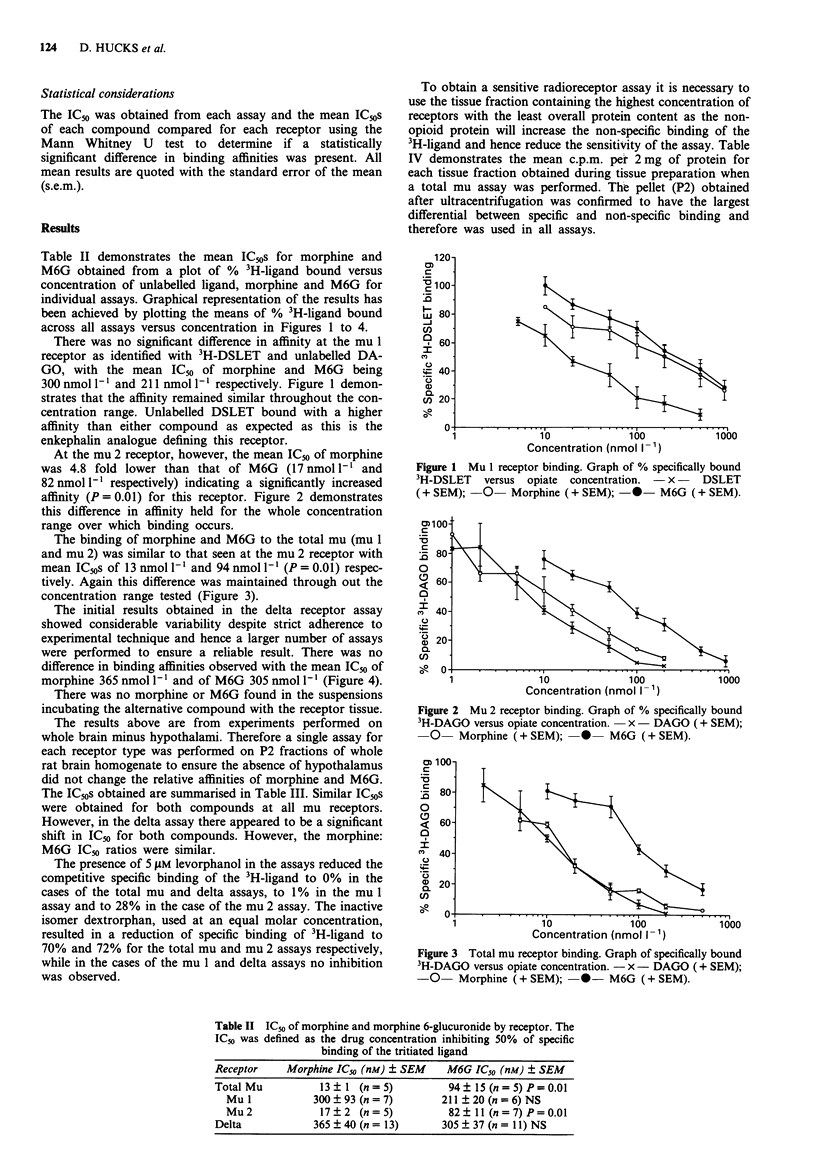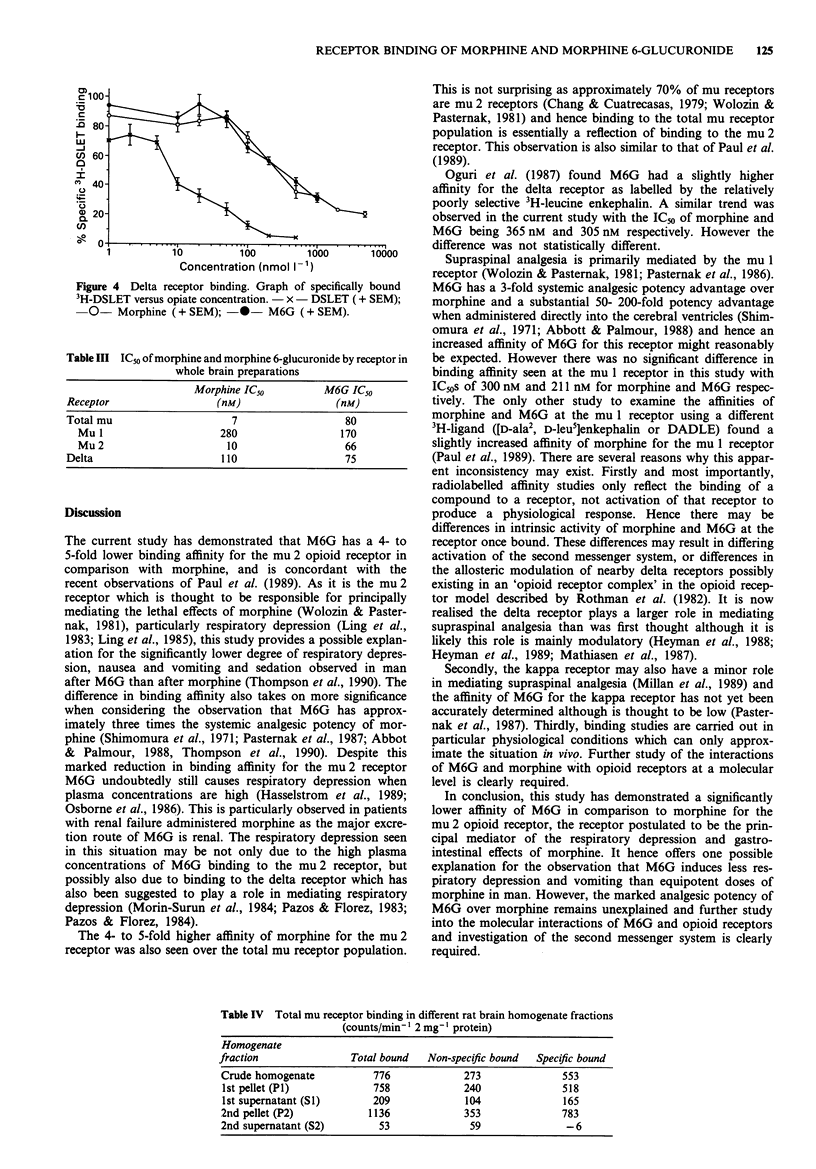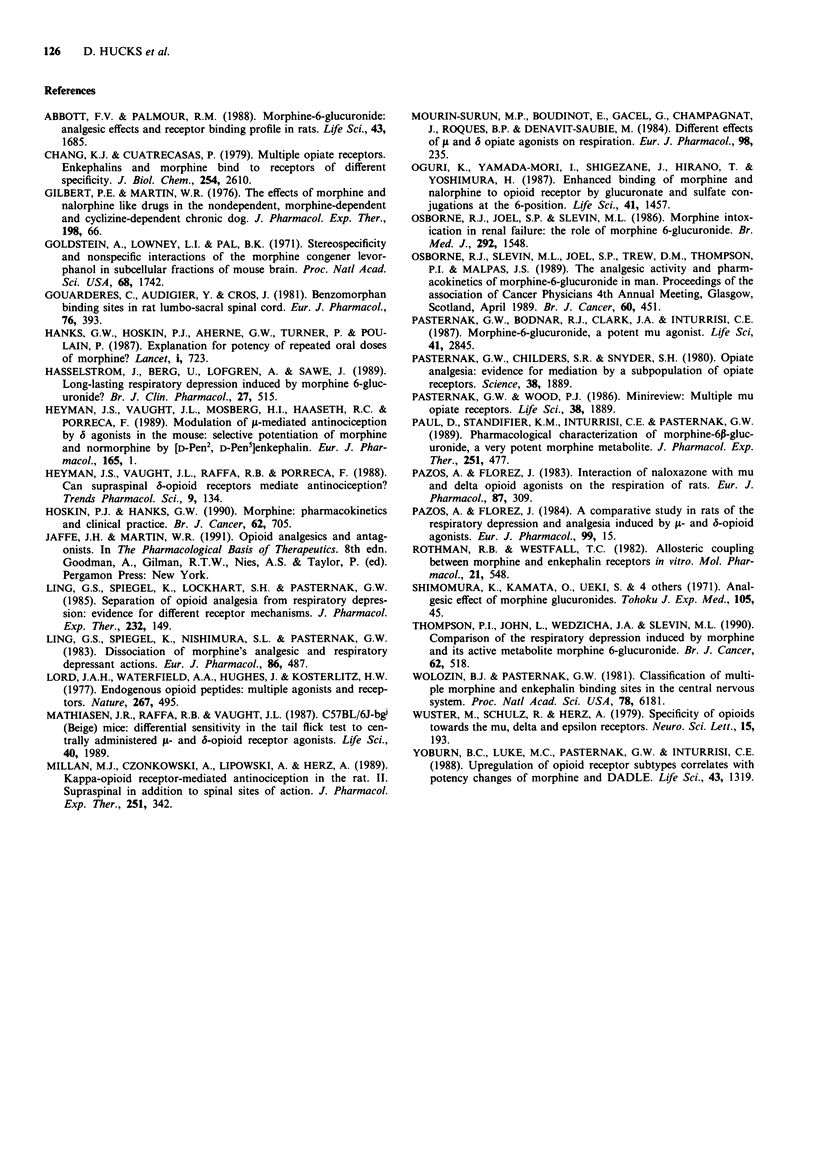# Explanation at the opioid receptor level for differing toxicity of morphine and morphine 6-glucuronide.

**DOI:** 10.1038/bjc.1992.23

**Published:** 1992-01

**Authors:** D. Hucks, P. I. Thompson, L. McLoughlin, S. P. Joel, N. Patel, A. Grossman, L. H. Rees, M. L. Slevin

**Affiliations:** Department of Chemical Endocrinology, St Bartholomew's Hospital, London, UK.

## Abstract

The radiolabelled opioid receptor binding affinities of morphine and its active metabolite morphine 6-glucuronide at the total mu, mu 1, mu 2 and delta receptors were determined. Morphine 6-glucuronide was found to have a 4-fold lower affinity for the mu 2 receptor (IC50 17 nM and 82 nM for morphine and morphine 6-glucuronide respectively, P = 0.01), the receptor postulated to be responsible for mediating the respiratory depression and gastrointestinal effects after morphine. This provides a possible explanation for the reduced respiratory depression and vomiting seen following morphine 6-glucuronide in man. A similar reduction in affinity of morphine 6-glucuronide was seen at the total mu receptor whilst there was no significant difference seen at the mu 1 or delta receptor. Hence the increased analgesic potency of morphine 6-glucuronide over morphine remains unexplained.


Br. J. Cancer (1992), 65, 122–126 © Macmillan Press Ltd., 1992

### Explanation at the opioid receptor level for differing toxicity of morphine and morphine 6-glucuronide

D. Hucks[1], P.I. Thompson[2], L. McLoughlin[1], S.P. Joel[2], N. Patel[2], A. Grossman[1], L.H. Rees[1] & M.L. Slevin[2]

[1]*Department of Chemical Endocrinology, St Bartholomew's Hospital, London;* [2]*ICRF Department of Medical Oncology, St Bartholomew's and Homerton Hospitals, London, UK.*

**Summary** The radiolabelled opioid receptor binding affinities of morphine and its active metabolite morphine 6-glucuronide at the total mu, mu 1, mu 2 and delta receptors were determined. Morphine 6-glucuronide was found to have a 4-fold lower affinity for the mu 2 receptor ($IC_{50}$ 17 nM and 82 nM for morphine and morphine 6-glucuronide respectively, $P = 0.01$), the receptor postulated to be responsible for mediating the respiratory depression and gastrointestinal effects after morphine. This provides a possible explanation for the reduced respiratory depression and vomiting seen following morphine 6-glucuronide in man. A similar reduction in affinity of morphine 6-glucuronide was seen at the total mu receptor whilst there was no significant difference seen at the mu 1 or delta receptor. Hence the increased analgesic potency of morphine 6-glucuronide over morphine remains unexplained.

Morphine is one of the commonest drugs prescribed by cancer physicians and is an effective potent analgesic. However one or more of the side effects of constipation, nausea and vomiting, and sedation are encountered frequently (Jaffe & Martin, 1991). Respiratory depression is a less common problem but is the most potentially dangerous toxicity. An analgesic with equivalent potency but lower toxicity would therefore be of particular use.

The major metabolic products of morphine are morphine 3-glucuronide (M3G) and morphine 6-glucuronide (M6G). Although M3G is devoid of analgesic activity, M6G is now thought to play a major role in mediating the analgesic effect of morphine (Osborne *et al.*, 1986; Hanks *et al.*, 1987; Hoskin & Hanks, 1990). When given directly M6G has been demonstrated to have more potent antinociceptive activity than morphine in animals (Shimomura *et al.*, 1971; Pasternak *et al.*, 1987; Abbott & Palmour, 1988; Paul *et al.*, 1989). It is tempting to presume the receptor binding profile of morphine and M6G is similar with M6G purely binding more avidly to the same receptors as morphine. However there are now several pieces of biochemical and clinical evidence to suggest this is not the case. Shimomum *et al.* (1971) found the systemic $LD_{50}$ of M6G to be 88% that of morphine and hence first suggested the lethal effects of M6G did not directly correlate with its analgesic potency advantage over morphine. In addition glucuronidation is normally a natural mechanism of the body to detoxify noxious substances.

An open study in man (Osborne *et al.*, 1989) provided anecdotal evidence in man that M6G may have a better toxicity profile than morphine as no nausea or sedation was seen at doses sufficient to give significant pain relief. A recent double-blind randomised study comparing the respiratory depression induced by equipotent doses of morphine and M6G in normal volunteers (Thompson *et al.*, 1990) demonstrated that significantly less respiratory depression was caused by M6G. In addition less nausea and no sedation was again observed.

The apparent improved therapeutic /toxic ratio of M6G over morphine suggests these compounds have different opioid receptor binding profiles. From a number of studies the existence of several types of opioid receptor has been proposed (Gilbert & Martin, 1976; Lord *et al.*, 1977; Wuster *et al.*, 1979; Wolozin *et al.*, 1981; Pasternak *et al.*, 1980. Gouarderes *et al.*, 1981; Rothman & Westfall, 1982), but there remains controversy over the existence and the functional and structural relationships of the various opioid receptor subtypes, particularly of the mu and delta receptor. Despite this, recent developments in producing selective enkephalin agonists has enabled the comparative affinity of different compounds to individual types of receptor to be determined via radioligand binding studies.

For the purpose of the current study we have chosen the model proposed first by Pasternak and colleagues (Wolozin & Pasternak, 1981). This model suggests there is a common receptor labelled by either a prototypic delta agonist such as DADLE (D-Ala$^2$, D-Leu$^5$-enkephalin) or with a mu agonist such as morphine which has high affinity for morphine. This they termed the mu 1 receptor. The receptor labelled with a mu agonist which possessed a lower affinity they termed the mu 2 receptor. Similarly the receptor labelled with a delta agonist possessing lower affinity for morphine they termed the true delta receptor.

It has been postulated that several of the adverse affects including respiratory depression of morphine are due to activation of the mu 2 or lower affinity mu opioid receptor (Pasternak & Wood, 1986). Using this classification it was therefore hypothesised that M6G has a lower affinity for the mu 2 receptor than morphine at least partially explaining the lower apparent toxicity seen in man. The aim of the current study was to investigate this hypothesis by comparing the receptor affinities of the two compounds by the use of radiolabelled binding studies on homogenised rat brain preparations.

### Materials and methods

In this study the method of Yoburn *et al.* (1988) was adapted to provide binding affinities, as expressed by the $IC_{50}$, of morphine and M6G at each of the mu 1, mu 2, total mu and delta receptors. The $IC_{50}$ is defined as the concentration of morphine or M6G to displace 50% of the $^3$H-ligand specifically bound to the opioid receptor subtype.

### *Tissue preparation*

Binding studies were performed using homogenised brain (minus the hypothalami used by other investigators) preparations from Wistar rats weighing 200–300 g. The rats were decapitated, the brains removed and hypothalami dissected out, before being washed in ice-cold 10 mM Tris HCl (pH 7.4 at room temperature), damped dry and weighed. The brains were then minced with scissors and homogenised in 4 × w/v

Correspondence: P.I. Thompson, Department of Clinical Oncology, Auckland Hospital, Park Road, Grafton, Auckland, 1, New Zealand.
Received 25 April 1991; and in revised form 5 September 1991.

ice-cold 10 mM HCl containing 0.32 M sucrose (Goldstein *et al.*, 1971) using an Ultra Turrex tissue homogeniser (Janke and Kankel Ltd). The homogenates were kept standing on ice throughout the procedure. Homogenates were centrifuged at 1000 *g* for 10 min at 4°C to remove the crude nuclear pellet. The supernatant was removed and recentrifuged at 100,000 *g* for 90 min at 4°C. The supernatant was removed and the pellet (P2) resuspended in a minimal volume of 10 mM Tris HCl buffer. An estimation of the protein concentration was made by using a modified Lowry protein assay (Boerhinger Mannheim, Mk78). The P2 suspension was then diluted with buffer to achieve a final protein concentration of 4 mg ml$^{-1}$. The P2 fractions were aliquoted and stored at −30°C for up to 3 weeks.

*Peptides*

DAGO, DSLET and DPDPE were obtained from Peninsula laboratories and $^3$H-DAGO and $^3$H-DSLET from Du Pont (UK) Ltd. Morphine sulphate was obtained from McCarthy Medical Ltd, UK, and M6G from Ultrafine Chemicals Ltd, UK.

*Assay procedure*

The list of synthetic enkephalin ligands used with their assumed receptor specifities is displayed in Table I. All assays for specific binding were performed in triplicate in 10 ml plastic test tubes and assays for total binding and non-specific binding performed in quadruplicate using the following experimental procedure:

*Total bindings* (i) 0.5 ml P2 (4 mg ml$^{-1}$).
(ii) 0.1 ml $^3$H-DSLET (1.5 nM) (mu 1 and delta assays) or 0.1 ml $^3$H-DAGO (1.5 nM) (total mu and mu 2 assays).
(iii) 0.1 ml Tris-HCl buffer (pH 7.4 at room temperature).
Vortex and pre-incubate in shaking water bath at 37°C for 5 min.
(iv) 0.1 ml Tris-HCl buffer.
Vortex and incubate in shaking water bath at 37°C for a further 15 min.

*Non-specific binding* (i) 0.5 ml P2 (4 mg ml$^{-1}$).
(ii) 0.1 ml $^3$H-DSLET (1.5 nM) (mu 1 and delta assays) or 0.1 ml $^3$H-DAGO (1.5 nM) (for total mu and mu 2 assays).
(iii) 0.1 ml DPDPE (20 nM) (mu 1 assay), 0.1 ml DSLET (5 nM) (mu 2 assay), 0.1 ml DAGO (5 nM) (delta assay) or 0.1 ml Tris-HCl buffer (total mu assay).
Vortex and pre-incubate in shaking water bath at 37°C for 5 min.
(iv) 0.1 ml DSLET (1 μM) (mu 1 assay), 0.1 ml DAGO (1 μM) (total mu and mu 2 assays) or 0.1 ml DSLET (1 μM) (delta assay).
Vortex and incubate in shaking water bath at 37°C for a further 15 min.

*Specific binding* (i) 0.5 ml P2 (4 mg ml$^{-1}$).
(ii) 0.1 ml $^3$H-DAGO (1.5 nM) (total mu and mu 2 assays) or 0.1 ml $^3$H-DSLET (1.5 nM) (mu 1 and delta assays).
(iii) 0.1 ml Tris-HCl buffer (total mu assay), 0.1 ml DPDPE (20 nM) (mu 1 assay), 0.1 ml DSLET (5 nM) (mu 2 assay) or 0.1 ml DAGO (5 nM) (delta assay).
Vortex and pre-incubate in shaking water bath at 37°C for 5 min.
(iv) 0.1 ml DSLET (mu 1 and delta assays), DAGO (total mu and mu 2 assays), morphine or M6G standards at concentrations specified below, or 0.1 ml Tris-HCl buffer.
Vortex and incubate in shaking water bath at 37°C for 15 min.

Following the second incubation the procedure for all assays was identical. The reaction was stopped by the addition of 5 ml ice cold Tris-HCl buffer to all tubes. The resulting solution was filtered through Whatman GF/B glass fibre filters using a Baker 10 extraction system capable of filtering ten samples simultaneously. The filter was washed twice with 5 ml of 10 mM Tris-HCl buffer and placed in scintillation bottles with 5 ml scintillant fluid (Ecoscint, National Diagnostics). Four unused filter papers were impregnated with 0.1 ml of the relevant $^3$H-ligand and added to four similar vials for the calculation of total counts. All vials are stored for 48 h prior to counting in a Tri-care liquid scintillation counter model 2425 (Hewlett Packard).

*Standards* For all assays blocking agent concentrations were based on those suggested from studies of Yoburn *et al.* (1988). The concentration range of opiate standards ranged from zero, which gave 100% specific binding, to the concentration required which inhibited all specific binding of $^3$H-ligand to the opioid receptor (binding equivalent to the NSB).

*Validation procedures* To confirm there was no biotransformation of morphine to M6G or vice-versa whilst incubation was in progress, after incubation some tubes were assayed by HPLC assessing morphine and M6G concentrations. In addition, to confirm the binding was actually at the opioid receptor, all assays were performed in the presence or absence of levorphanol (5 μM) or its inactive stereoisomer dextrorphan (5 μM). In order to determine that the P2 was the optimal fraction for use in the binding assays, all fractions produced during tissue preparations (P1, S1, P2 and S2) were assessed for opioid binding activity by their ability to bind the $^3$H-DAGO ligand.

*Calculation of results*

The mean counts from the three or four tubes were calculated prior to the calculation of binding.

The results are expressed as the percentage of specifically bound $^3$H-ligand *vs* concentration of opioid.

Non-specific binding was defined as counts in the presence of 1 μM non-radioactive enkephalin ligand.

$$\%\ \text{Total bound} = \frac{\text{counts of zero standard (std)} - \text{NSB}}{\text{total counts}} \times 100$$

$$\begin{array}{l}\%\ \text{Specific bound} \\ \text{(of total binding)}\end{array} = \frac{\text{counts of zero std} - \text{NSB}}{\text{counts of zero std}} \times 100$$

$$\%\ ^3\text{H-ligand bound} = \frac{\text{counts of std} - \text{NSB}}{\text{counts of zero std} - \text{NSB}} \times 100$$

From a plot of percentage $^3$H-ligand bound versus opiate concentration an $IC_{50}$ was ascertained and the $IC_{50}$s of cold ligand (DAGO or DSLET), morphine and M6G compared to determine relative binding affinities at each receptor.

**Table I** List of synthetic enkephalin ligands used and their receptor specificities

| *Synthetic ligand* | *Receptor specificity* |
|---|---|
| DAGO ([D-ala$^2$-MePhe$^4$, Gly-ol$^5$]-enkephalin) | mu 1 and mu 2 (total mu) |
| DSLET ([D-Ser$^2$, Leu$^5$]-enkephalin) | mu 1 and delta |
| DPDPE ([D-Pen$^2$, D-Pen$^5$]-enkephalin) | delta |

*Statistical considerations*

The $IC_{50}$ was obtained from each assay and the mean $IC_{50}$s of each compound compared for each receptor using the Mann Whitney U test to determine if a statistically significant difference in binding affinities was present. All mean results are quoted with the standard error of the mean (s.e.m.).

### Results

Table II demonstrates the mean $IC_{50}$s for morphine and M6G obtained from a plot of % $^3$H-ligand bound versus concentration of unlabelled ligand, morphine and M6G for individual assays. Graphical representation of the results has been achieved by plotting the means of % $^3$H-ligand bound across all assays versus concentration in Figures 1 to 4.

There was no significant difference in affinity at the mu 1 receptor as identified with $^3$H-DSLET and unlabelled DAGO, with the mean $IC_{50}$ of morphine and M6G being 300 nmol $l^{-1}$ and 211 nmol $l^{-1}$ respectively. Figure 1 demonstrates that the affinity remained similar throughout the concentration range. Unlabelled DSLET bound with a higher affinity than either compound as expected as this is the enkephalin analogue defining this receptor.

At the mu 2 receptor, however, the mean $IC_{50}$ of morphine was 4.8 fold lower than that of M6G (17 nmol $l^{-1}$ and 82 nmol $l^{-1}$ respectively) indicating a significantly increased affinity ($P = 0.01$) for this receptor. Figure 2 demonstrates this difference in affinity held for the whole concentration range over which binding occurs.

The binding of morphine and M6G to the total mu (mu 1 and mu 2) was similar to that seen at the mu 2 receptor with mean $IC_{50}$s of 13 nmol $l^{-1}$ and 94 nmol $l^{-1}$ ($P = 0.01$) respectively. Again this difference was maintained through out the concentration range tested (Figure 3).

The initial results obtained in the delta receptor assay showed considerable variability despite strict adherence to experimental technique and hence a larger number of assays were performed to ensure a reliable result. There was no difference in binding affinities observed with the mean $IC_{50}$ of morphine 365 nmol $l^{-1}$ and of M6G 305 nmol $l^{-1}$ (Figure 4).

There was no morphine or M6G found in the suspensions incubating the alternative compound with the receptor tissue.

The results above are from experiments performed on whole brain minus hypothalami. Therefore a single assay for each receptor type was performed on P2 fractions of whole rat brain homogenate to ensure the absence of hypothalamus did not change the relative affinities of morphine and M6G. The $IC_{50}$s obtained are summarised in Table III. Similar $IC_{50}$s were obtained for both compounds at all mu receptors. However, in the delta assay there appeared to be a significant shift in $IC_{50}$ for both compounds. However, the morphine: M6G $IC_{50}$ ratios were similar.

The presence of 5 μM levorphanol in the assays reduced the competitive specific binding of the $^3$H-ligand to 0% in the cases of the total mu and delta assays, to 1% in the mu 1 assay and to 28% in the case of the mu 2 assay. The inactive isomer dextrorphan, used at an equal molar concentration, resulted in a reduction of specific binding of $^3$H-ligand to 70% and 72% for the total mu and mu 2 assays respectively, while in the cases of the mu 1 and delta assays no inhibition was observed.

To obtain a sensitive radioreceptor assay it is necessary to use the tissue fraction containing the highest concentration of receptors with the least overall protein content as the non-opioid protein will increase the non-specific binding of the $^3$H-ligand and hence reduce the sensitivity of the assay. Table IV demonstrates the mean c.p.m. per 2 mg of protein for each tissue fraction obtained during tissue preparation when a total mu assay was performed. The pellet (P2) obtained after ultracentrifugation was confirmed to have the largest differential between specific and non-specific binding and therefore was used in all assays.

**Figure 1** Mu 1 receptor binding. Graph of % specifically bound $^3$H-DSLET versus opiate concentration. —×— DSLET (+SEM); —O— Morphine (+SEM); —●— M6G (+SEM).

**Figure 2** Mu 2 receptor binding. Graph of % specifically bound $^3$H-DAGO versus opiate concentration. —×— DAGO (+SEM); —O— Morphine (+SEM); —●— M6G (+SEM).

**Figure 3** Total mu receptor binding. Graph of specifically bound $^3$H-DAGO versus opiate concentration. —×— DAGO (+SEM); —O— Morphine (+SEM); —●— M6G (+SEM).

**Table II** $IC_{50}$ of morphine and morphine 6-glucuronide by receptor. The $IC_{50}$ was defined as the drug concentration inhibiting 50% of specific binding of the tritiated ligand

| *Receptor* | *Morphine $IC_{50}$ (nM) ± SEM* | *M6G $IC_{50}$ (nM) ± SEM* |
|---|---|---|
| Total Mu | 13 ± 1 (*n* = 5) | 94 ± 15 (*n* = 5) $P = 0.01$ |
| Mu 1 | 300 ± 93 (*n* = 7) | 211 ± 20 (*n* = 6) NS |
| Mu 2 | 17 ± 2 (*n* = 5) | 82 ± 11 (*n* = 7) $P = 0.01$ |
| Delta | 365 ± 40 (*n* = 13) | 305 ± 37 (*n* = 11) NS |

**Figure 4** Delta receptor binding. Graph of specifically bound $^3$H-DSLET versus opiate concentration. —×— DSLET (+SEM); —○— Morphine (+SEM); —●— M6G (+SEM).

**Table III** $IC_{50}$ of morphine and morphine 6-glucuronide by receptor in whole brain preparations

| Receptor | *Morphine $IC_{50}$ (nM)* | *M6G $IC_{50}$ (nM)* |
|---|---|---|
| Total mu | 7 | 80 |
| Mu 1 | 280 | 170 |
| Mu 2 | 10 | 66 |
| Delta | 110 | 75 |

### Discussion

The current study has demonstrated that M6G has a 4- to 5-fold lower binding affinity for the mu 2 opioid receptor in comparison with morphine, and is concordant with the recent observations of Paul *et al.* (1989). As it is the mu 2 receptor which is thought to be responsible for principally mediating the lethal effects of morphine (Wolozin & Pasternak, 1981), particularly respiratory depression (Ling *et al.*, 1983; Ling *et al.*, 1985), this study provides a possible explanation for the significantly lower degree of respiratory depression, nausea and vomiting and sedation observed in man after M6G than after morphine (Thompson *et al.*, 1990). The difference in binding affinity also takes on more significance when considering the observation that M6G has approximately three times the systemic analgesic potency of morphine (Shimomura *et al.*, 1971; Pasternak *et al.*, 1987; Abbot & Palmour, 1988, Thompson *et al.*, 1990). Despite this marked reduction in binding affinity for the mu 2 receptor M6G undoubtedly still causes respiratory depression when plasma concentrations are high (Hasselstrom *et al.*, 1989; Osborne *et al.*, 1986). This is particularly observed in patients with renal failure administered morphine as the major excretion route of M6G is renal. The respiratory depression seen in this situation may be not only due to the high plasma concentrations of M6G binding to the mu 2 receptor, but possibly also due to binding to the delta receptor which has also been suggested to play a role in mediating respiratory depression (Morin-Surun *et al.*, 1984; Pazos & Florez, 1983; Pazos & Florez, 1984).

The 4- to 5-fold higher affinity of morphine for the mu 2 receptor was also seen over the total mu receptor population. This is not surprising as approximately 70% of mu receptors are mu 2 receptors (Chang & Cuatrecasas, 1979; Wolozin & Pasternak, 1981) and hence binding to the total mu receptor population is essentially a reflection of binding to the mu 2 receptor. This observation is also similar to that of Paul *et al.* (1989).

Oguri *et al.* (1987) found M6G had a slightly higher affinity for the delta receptor as labelled by the relatively poorly selective $^3$H-leucine enkephalin. A similar trend was observed in the current study with the $IC_{50}$ of morphine and M6G being 365 nM and 305 nM respectively. However the difference was not statistically different.

Supraspinal analgesia is primarily mediated by the mu 1 receptor (Wolozin & Pasternak, 1981; Pasternak *et al.*, 1986). M6G has a 3-fold systemic analgesic potency advantage over morphine and a substantial 50- 200-fold potency advantage when administered directly into the cerebral ventricles (Shimomura *et al.*, 1971; Abbott & Palmour, 1988) and hence an increased affinity of M6G for this receptor might reasonably be expected. However there was no significant difference in binding affinity seen at the mu 1 receptor in this study with $IC_{50}$s of 300 nM and 211 nM for morphine and M6G respectively. The only other study to examine the affinities of morphine and M6G at the mu 1 receptor using a different $^3$H-ligand ([D-ala$^2$, D-leu$^5$]enkephalin or DADLE) found a slightly increased affinity of morphine for the mu 1 receptor (Paul *et al.*, 1989). There are several reasons why this apparent inconsistency may exist. Firstly and most importantly, radiolabelled affinity studies only reflect the binding of a compound to a receptor, not activation of that receptor to produce a physiological response. Hence there may be differences in intrinsic activity of morphine and M6G at the receptor once bound. These differences may result in differing activation of the second messenger system, or differences in the allosteric modulation of nearby delta receptors possibly existing in an 'opioid receptor complex' in the opioid receptor model described by Rothman *et al.* (1982). It is now realised the delta receptor plays a larger role in mediating supraspinal analgesia than was first thought although it is likely this role is mainly modulatory (Heyman *et al.*, 1988; Heyman *et al.*, 1989; Mathiasen *et al.*, 1987).

Secondly, the kappa receptor may also have a minor role in mediating supraspinal analgesia (Millan *et al.*, 1989) and the affinity of M6G for the kappa receptor has not yet been accurately determined although is thought to be low (Pasternak *et al.*, 1987). Thirdly, binding studies are carried out in particular physiological conditions which can only approximate the situation *in vivo*. Further study of the interactions of M6G and morphine with opioid receptors at a molecular level is clearly required.

In conclusion, this study has demonstrated a significantly lower affinity of M6G in comparison to morphine for the mu 2 opioid receptor, the receptor postulated to be the principal mediator of the respiratory depression and gastro-intestinal effects of morphine. It hence offers one possible explanation for the observation that M6G induces less respiratory depression and vomiting than equipotent doses of morphine in man. However, the marked analgesic potency of M6G over morphine remains unexplained and further study into the molecular interactions of M6G and opioid receptors and investigation of the second messenger system is clearly required.

**Table IV** Total mu receptor binding in different rat brain homogenate fractions (counts/min$^{-1}$ 2 mg$^{-1}$ protein)

| *Homogenate fraction* | *Total bound* | *Non-specific bound* | *Specific bound* |
|---|---|---|---|
| Crude homogenate | 776 | 273 | 553 |
| 1st pellet (P1) | 758 | 240 | 518 |
| 1st supernatant (S1) | 209 | 104 | 165 |
| 2nd pellet (P2) | 1136 | 353 | 783 |
| 2nd supernatant (S2) | 53 | 59 | −6 |

### References

ABBOTT, F.V. & PALMOUR, R.M. (1988). Morphine-6-glucuronide: analgesic effects and receptor binding profile in rats. *Life Sci.*, **43**, 1685.

CHANG, K.J. & CUATRECASAS, P. (1979). Multiple opiate receptors. Enkephalins and morphine bind to receptors of different specificity. *J. Biol. Chem.*, **254**, 2610.

GILBERT, P.E. & MARTIN, W.R. (1976). The effects of morphine and nalorphine like drugs in the nondependent, morphine-dependent and cyclizine-dependent chronic dog. *J. Pharmacol. Exp. Ther.*, **198**, 66.

GOLDSTEIN, A., LOWNEY, L.I. & PAL, B.K. (1971). Stereospecificity and nonspecific interactions of the morphine congener levorphanol in subcellular fractions of mouse brain. *Proc. Natl Acad. Sci. USA*, **68**, 1742.

GOUARDERES, C., AUDIGIER, Y. & CROS, J. (1981). Benzomorphan binding sites in rat lumbo-sacral spinal cord. *Eur. J. Pharmacol.*, **76**, 393.

HANKS, G.W., HOSKIN, P.J., AHERNE, G.W., TURNER, P. & POULAIN, P. (1987). Explanation for potency of repeated oral doses of morphine? *Lancet*, **i**, 723.

HASSELSTROM, J., BERG, U., LOFGREN, A. & SAWE, J. (1989). Long-lasting respiratory depression induced by morphine 6-glucuronide? *Br. J. Clin. Pharmacol.*, **27**, 515.

HEYMAN, J.S., VAUGHT, J.L., MOSBERG, H.I., HAASETH, R.C. & PORRECA, F. (1989). Modulation of $\mu$-mediated antinociception by $\delta$ agonists in the mouse: selective potentiation of morphine and normorphine by [D-Pen$^2$, D-Pen$^5$]enkephalin. *Eur. J. Pharmacol.*, **165**, 1.

HEYMAN, J.S., VAUGHT, J.L., RAFFA, R.B. & PORRECA, F. (1988). Can supraspinal $\delta$-opioid receptors mediate antinociception? *Trends Pharmacol. Sci.*, **9**, 134.

HOSKIN, P.J. & HANKS, G.W. (1990). Morphine: pharmacokinetics and clinical practice. *Br. J. Cancer*, **62**, 705.

JAFFE, J.H. & MARTIN, W.R. (1991). Opioid analgesics and antagonists. In *The Pharmacological Basis of Therapeutics*. 8th edn. Goodman, A., Gilman, R.T.W., Nies, A.S. & Taylor, P. (ed). Pergamon Press: New York.

LING, G.S., SPIEGEL, K., LOCKHART, S.H. & PASTERNAK, G.W. (1985). Separation of opioid analgesia from respiratory depression: evidence for different receptor mechanisms. *J. Pharmacol. Exp. Ther.*, **232**, 149.

LING, G.S., SPIEGEL, K., NISHIMURA, S.L. & PASTERNAK, G.W. (1983). Dissociation of morphine's analgesic and respiratory depressant actions. *Eur. J. Pharmacol.*, **86**, 487.

LORD, J.A.H., WATERFIELD, A.A., HUGHES, J. & KOSTERLITZ, H.W. (1977). Endogenous opioid peptides: multiple agonists and receptors. *Nature*, **267**, 495.

MATHIASEN, J.R., RAFFA, R.B. & VAUGHT, J.L. (1987). C57BL/6J-bg$^j$ (Beige) mice: differential sensitivity in the tail flick test to centrally administered $\mu$- and $\delta$-opioid receptor agonists. *Life Sci.*, **40**, 1989.

MILLAN, M.J., CZONKOWSKI, A., LIPOWSKI, A. & HERZ, A. (1989). Kappa-opioid receptor-mediated antinociception in the rat. II. Supraspinal in addition to spinal sites of action. *J. Pharmacol. Exp. Ther.*, **251**, 342.

MOURIN-SURUN, M.P., BOUDINOT, E., GACEL, G., CHAMPAGNAT, J., ROQUES, B.P. & DENAVIT-SAUBIE, M. (1984). Different effects of $\mu$ and $\delta$ opiate agonists on respiration. *Eur. J. Pharmacol.*, **98**, 235.

OGURI, K., YAMADA-MORI, I., SHIGEZANE, J., HIRANO, T. & YOSHIMURA, H. (1987). Enhanced binding of morphine and nalorphine to opioid receptor by glucuronate and sulfate conjugations at the 6-position. *Life Sci.*, **41**, 1457.

OSBORNE, R.J., JOEL, S.P. & SLEVIN, M.L. (1986). Morphine intoxication in renal failure: the role of morphine 6-glucuronide. *Br. Med. J.*, **292**, 1548.

OSBORNE, R.J., SLEVIN, M.L., JOEL, S.P., TREW, D.M., THOMPSON, P.I. & MALPAS, J.S. (1989). The analgesic activity and pharmacokinetics of morphine-6-glucuronide in man. Proceedings of the association of Cancer Physicians 4th Annual Meeting, Glasgow, Scotland, April 1989. *Br. J. Cancer*, **60**, 451.

PASTERNAK, G.W., BODNAR, R.J., CLARK, J.A. & INTURRISI, C.E. (1987). Morphine-6-glucuronide, a potent mu agonist. *Life Sci*, **41**, 2845.

PASTERNAK, G.W., CHILDERS, S.R. & SNYDER, S.H. (1980). Opiate analgesia: evidence for mediation by a subpopulation of opiate receptors. *Science*, **38**, 1889.

PASTERNAK, G.W. & WOOD, P.J. (1986). Minireview: Multiple mu opiate receptors. *Life Sci.*, **38**, 1889.

PAUL, D., STANDIFIER, K.M., INTURRISI, C.E. & PASTERNAK, G.W. (1989). Pharmacological characterization of morphine-6$\beta$-glucuronide, a very potent morphine metabolite. *J. Pharmacol. Exp. Ther.*, **251**, 477.

PAZOS, A. & FLOREZ, J. (1983). Interaction of naloxazone with mu and delta opioid agonists on the respiration of rats. *Eur. J. Pharmacol.*, **87**, 309.

PAZOS, A. & FLOREZ, J. (1984). A comparative study in rats of the respiratory depression and analgesia induced by $\mu$- and $\delta$-opioid agonists. *Eur. J. Pharmacol.*, **99**, 15.

ROTHMAN, R.B. & WESTFALL, T.C. (1982). Allosteric coupling between morphine and enkephalin receptors *in vitro*. *Mol. Pharmacol.*, **21**, 548.

SHIMOMURA, K., KAMATA, O., UEKI, S. & 4 others (1971). Analgesic effect of morphine glucuronides. *Tohoku J. Exp. Med.*, **105**, 45.

THOMPSON, P.I., JOHN, L., WEDZICHA, J.A. & SLEVIN, M.L. (1990). Comparison of the respiratory depression induced by morphine and its active metabolite morphine 6-glucuronide. *Br. J. Cancer*, **62**, 518.

WOLOZIN, B.J. & PASTERNAK, G.W. (1981). Classification of multiple morphine and enkephalin binding sites in the central nervous system. *Proc. Natl Acad. Sci. USA*, **78**, 6181.

WUSTER, M., SCHULZ, R. & HERZ, A. (1979). Specificity of opioids towards the mu, delta and epsilon receptors. *Neuro. Sci. Lett.*, **15**, 193.

YOBURN, B.C., LUKE, M.C., PASTERNAK, G.W. & INTURRISI, C.E. (1988). Upregulation of opioid receptor subtypes correlates with potency changes of morphine and DADLE. *Life Sci.*, **43**, 1319.